# Influence of phosphate dosing on biofilms development on lead in chlorinated drinking water bioreactors

**DOI:** 10.1038/s41522-020-00152-w

**Published:** 2020-10-23

**Authors:** Gonzalo Del Olmo, Arslan Ahmad, Henriette Jensen, Esther Karunakaran, Esther Rosales, Carolina Calero Preciado, Paul Gaskin, Isabel Douterelo

**Affiliations:** 1 Department of Civil and Structural Engineering, University of Sheffield, Sheffield, UK; 2 KWR Water Cycle Research Institute, Groningenhaven 7, 3433 PE Nieuwegein, The Netherlands; 3 KTH-International Groundwater Arsenic Research Group, Department of Sustainable Development, Environmental Science and Engineering, KTH Royal Institute of Technology, Teknikringen 10B, SE-100 44 Stockholm, Sweden; 4 Department of Environmental Technology, Wageningen University and Research (WUR), Droevendaalsesteeg 4, 6708 PB Wageningen, The Netherlands; 5 Department of Chemical and Biological Engineering, University of Sheffield, Sheffield, UK; 6 Dwr Cymru Welsh Water, Newport, UK

**Keywords:** Biofilms, Metagenomics

## Abstract

Phosphate dosing is used by water utilities to prevent plumbosolvency in water supply networks. However, there is a lack of knowledge regarding biofilm formation on lead and plastic materials when phosphate concentrations are modified in drinking water systems. In this study, biofilms were grown over lead coupons and PVC tubes in bioreactors supplied with local drinking water treated to provide different phosphate doses (below 1, 1 and 2 mg/L) over a period of 28 days. A range of commercial iron pellets (GEH104 and WARP) were tested aiming to maintain phosphate levels below the average 1 mg/L found in drinking water. Changes in biofilm community structure in response to three different phosphate treatments were characterised by Illumina sequencing of the 16S rRNA gene for bacteria and the ITS2 gene for fungi. Scanning electron microscopy was used to visualise physical differences in biofilm development in two types of materials, lead and PVC. The experimental results from the kinetics of phosphate absorption showed that the GEH104 pellets were the best option to, in the long term, reduce phosphate levels while preventing undesirable turbidity increases in drinking water. Phosphate-enrichment promoted a reduction of bacterial diversity but increased that of fungi in biofilms. Overall, higher phosphate levels selected for microorganisms with enhanced capabilities related to phosphorus metabolism and heavy metal resistance. This research brings new insights regarding the influence of different phosphate concentrations on mixed-species biofilms formation and drinking water quality, which are relevant to inform best management practices in drinking water treatment.

## Introduction

Lead has been commonly used in building water supply infrastructure given its ductile properties and easy moulding to make pipes. However, lead is a heavy metal that can be toxic for organisms since it accumulates in the food chain and remains as a persistent environmental toxin^1^. Therefore, lead is considered the second most dangerous environmental poison according to the Agency for Toxic Substances and Disease Registry Priority Substance List (2017). Its toxic effects have been related to neurotoxicity, carcinogenicity, reproductive toxicology, renal failure, anaemia or cardiac dysfunctions, being children the age group with higher risk for lead poisoning^2^.

Lead can enter in drinking water distribution systems (DWDS) through leaching from lead pipes and other plumbing fittings^3^. Many DWDS in the world have lead pipes, including the United Kingdom (UK), where lead can be prevalent in properties built before 1970, and it has been estimated that 9 million households are affected by lead pipes^4^. Currently, the European Parliament establishes that the legal level of lead content in drinking water is 10 μg/L, and it is aiming to reduce this concentration to 5 μg/L in the following 10 years (COM(2017)0753—C8-0019/2018—2017/0332(COD)). Since the 1990s most of the UK’s public water supplies are being dosed with orthophosphate acid or a sodium orthophosphate to minimise plumbosolvency^5–7^, using concentrations ranging from 0.5 to 2 mg/L of phosphate (PO_4_^3−^)^6^. The different PO_4_^3−^ species are able to interact with the soluble lead ions (Pb^2+^), to form a layer of lead phosphate compounds which covers the inner side of the pipe^8–10^. Cities like Brussels or The Hague have chosen the costly option of substituting lead pipes in their distribution networks^4^, yet using PO_4_^3−^ is a successful and cost-effective alternative to spending ~£10 billion to remove all lead pipes in the UK^7^.

In DWDS, more than 95% of the biomass contained in these systems are microorganisms attached to structures forming biofilms^11^. In DWDS, biofilms have been associated with processes that might alter water quality such as metal bio-corrosion^12^, discolouration events^12,13^, pathogens appearance^12,13^ and lead accumulation and release^14–17^. Therefore, the consequences that PO_4_^3−^ has on biofilm formation must be considered when used in plumbosolvency treatment^15^.

To what extent PO_4_^3−^ affects the microbial ecology of DWDS remains unknown and previous research provides contradictory results. Several studies conclude that PO_4_^3−^ promoted the development of planktonic microorganisms in drinking water, and changed the bacterial community structure increasing Gram-negative bacterial content^18^. In relation to biofilm development on a range of substrates, it has been reported that PO_4_^3−^ increased the number of bacterial cells, promoted biofilm viability^19^ and diversity^20,21^, but reduced the production of exopolysaccharides^22^. In contrast, other studies indicated that the bacterial density was not affected by PO_4_^3−^ ^23^ and even a decrease of heterotrophic bacteria was observed^24^.

In relation to lead, there are only few studies focusing on the study of microbial communities growing on this type of metal^15–17,20,25^ and results from these suggested that lead did not affect biofilm biomass^20^, richness^20^ and bacterial numbers^17^, yet exerted a selective pressure on biofilm communities by favouring bacteria able to accumulate and resist heavy metals^15,17,20^.

In the UK, water utilities are moving towards reducing the costly use of chemical additives, including PO_4_^3−^, but this is currently constrained by the limited knowledge on the potential consequences that this might have down the pipeline. Therefore, to study the impact of phosphate reduction on the microbiome of DWDS and inform future management practices we aimed to remove PO_4_^3−^ from local drinking water. Most of the studies on phosphate removal have been performed to remove this chemical from wastewater effluents; however, there is limited knowledge on phosphate removal in drinking water. Previous research has shown that granular ferric hydroxide is effective at adsorbing and maintaining low concentrations of phosphate in water^26–30^. Considering this information, we tested for the first time the capability of two commercial iron pellets (GEH104 and WARP) for removal of phosphate in drinking water.

The aim of this study was to provide better insights into the effect of PO_4_^3−^ dosing on the development of mixed-species biofilms on lead and PVC surfaces in drinking water systems. With this aim, different doses of PO_4_^3−^ were used to develop biofilms under three conditions: (i) control condition: UK standard drinking water PO_4_^3−^ concentrations (1 mg/L of PO_4_^3−^ as phosphorus), (ii) high phosphate condition: double dose of the standard concentrations (2 mg/L of PO_4_^3−^ as phosphorus) and (iii) low phosphate condition: PO_4_^3−^ limited concentration below 1 mg/L.

To our knowledge, this is the first study that provides information about the effect of PO_4_^3−^ on mixed-species biofilms developed on lead and PVC materials in drinking water and aims to improve management strategies of DWDS. The data will inform water companies on the consequences of PO_4_^3−^ dosing in DWDS. This will impact drinking water network management decisions for (i) maintaining water quality standards by controlling microbial growth, (ii) facilitate changes in dose management, (iii) reduce chemical dosing costs, (iv) reduce downstream PO_4_^3−^ removal costs and (v) reduce environmental impact of PO_4_^3−^ leaking from ageing water infrastructure and treated water discharge.

## Results

### Kinetics of iron pellets to remove phosphate from drinking water

**Fig. 1:**
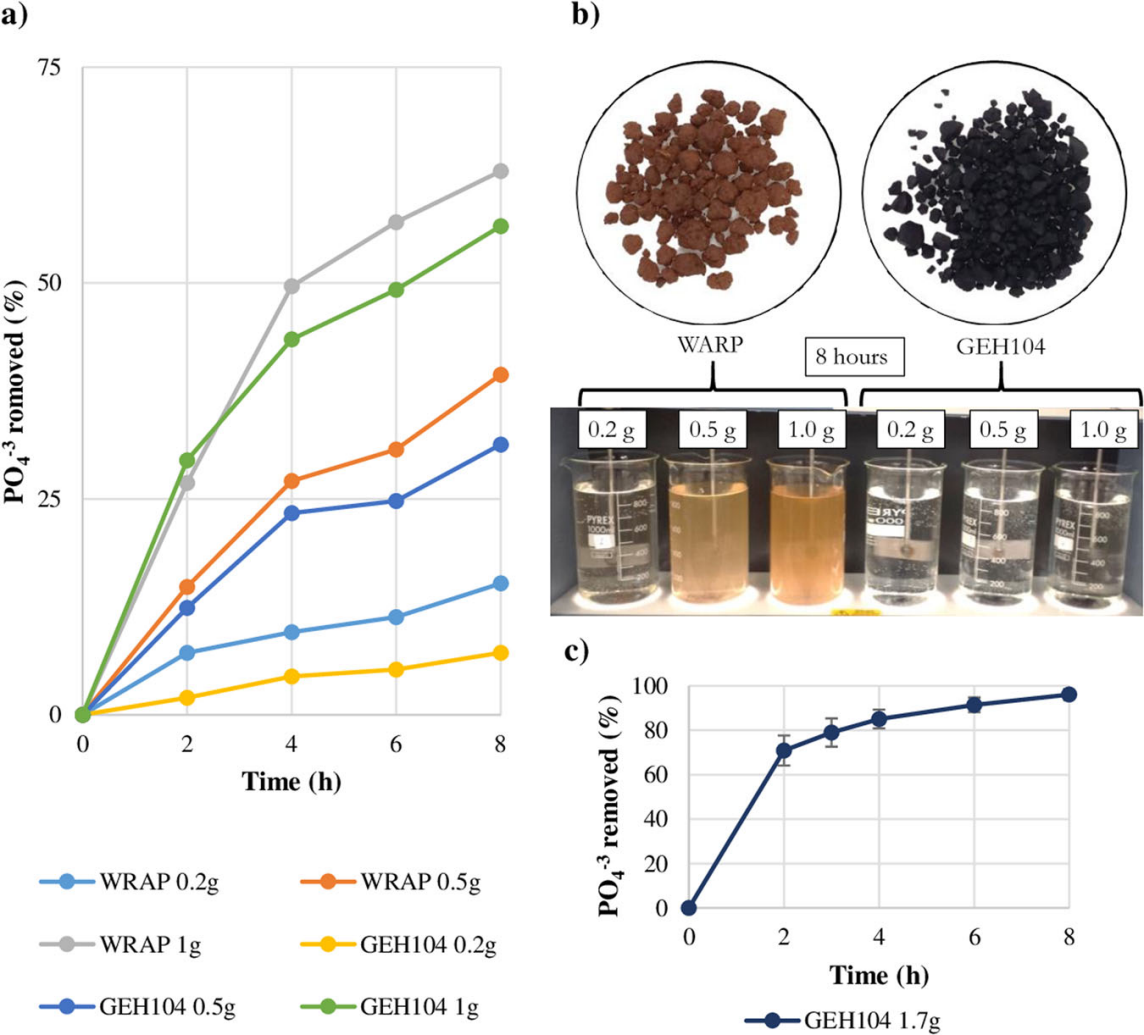
Iron pellets adsorption kinetics. **a** Comparison of phosphate removal capacity of iron pellets from 1 L of drinking water using three amounts (0.2, 0.5 and 1.0 g) of each type of pellets (WARP and GEH104) during 8 h. **b** Aspect of the drinking water in the presence of the two different amounts of iron pellets (WARP and GEH104) after 8 h. **c** Phosphate removal from drinking water with 1.7 g of GEH104 iron pellets during 8 h, the average and the standard deviations (error bars) of three replicas for each sample taken are shown.

Both types of pellets, WRAP and GEH104, absorbed PO_4_^3−^ successfully (Fig. 1a). There was a proportional relationship between the quantity of pellets added and the PO_4_^3−^ removed. With the higher amount of PO_4_^3−^ used (1 g/L), the pellets WRAP and GEH104 removed 63% and 57%, respectively. However, GEH104 generated less turbidity than WARP (Fig. 1b). Therefore, GEH104 were the pellets selected to run the experiments to test the effect of low PO_4_^3−^ on biofilm development in the bioreactors. Due to the proportional relationship between the amount of pellet and the PO_4_^3−^ removal rate, it was estimated that with 1.7 g/L of GEH104 added to the water, most of the PO_4_^3−^ would be removed during the 28-days experiment. To verify these calculations, a second experiment with 1.7 g of GEH104 pellets and 1 L of local drinking water was run for 8 h and in triplicate. These results showed that 1.7 g of GEH104 pellets could remove 96.08 ± 2.48% of PO_4_^3−^ in drinking water (Fig. 1c). Therefore, for the experiment in the bioreactors, a superior concentration of 2 g/L of GEH104 was used.

### Water physicochemical analysis: bioreactors and local drinking water supply

The results of water quality parameters measured in the three bioreactors and the local drinking water supply used for the experiments are summarised in Table 1. The orthophosphate content in water was stable in the three experimental cases (high phosphate: 2.48 ± 0.25 mg as P/L, control phosphate: 1.09 ± 0.09 mg as P/L and low phosphate: 0.03 ± 0.09 mg as P/L) and in local drinking water (1.12 ± 0.17 mg as P/L). Total organic carbon (TOC) did not experiment high fluctuations during the experiment, being lower in the case of low phosphate (0.95 ± 0.16 mg C/L) comparing with the other two cases (high phosphate: 1.41 ± 0.13 mg C/L and control phosphate: 1.47 ± 0.08 mg C/L) and the local drinking water (1.46 ± 0.14 mg C/L). Turbidity, lead and iron content in the local drinking water used to supply the system were very low (0.10 ± 0.05 µg Pb/L 12.47 ± 11.63 µg Fe/L and 0.26 ± 0.22 NTU). When the water in the bioreactors was analysed these three parameters were lower under high phosphate conditions (5.51 ± 2.54 µg Pb/L, 118.43 ± 27.51 µg Fe/L and 1.17 ± 0.33 NTU) than in the other two PO_4_^3−^ conditions (control phosphate: 7.75 ± 3.53 µg Pb/L, 239.07 ± 147.97 µg Fe/L and 1.57 ± 0.94 NTU, and low phosphate: 29.64 ± 11.95 µg Pb/L, 334.53 ± 62.52 µg Fe/L and 3.70 ± 1.76 NTU). On average, the water temperature remained stable for the three conditions studied during the duration of the experiment (high phosphate: 20.18 ± 1.76 °C, control phosphate: 20.66 ± 1.80 °C and low phosphate: 21.18 ± 1.43 °C), but it was lower in the local drinking water supplying the system (16.61 ± 2.29 °C).

**Table 1:** Physico-chemist parameters of water.

Sample	Time (day)	Free chlorine (mg/l)	Total chlorine (mg/l)	Turbidity (NTU)	pH	Room temperature (°C)	Water temperature (°C)	Iron, total as Fe (µg/l)	Lead, total as Pb (µg/l)	Orthophosphate as P (mg/l)	Phosphorus, total as P (mg/l)	Total organic carbon (mg/l)
High phosphate bioreactor	0	0.35 ± 0.06	0.45 ± 0.09	1.64 ± 0.76	6.75 ± 0.01	17.33 ± 0.10	15.60 ± 0.00	114.00 ± 2.65	9.63 ± 0.16	2.34 ± 0.04	2.24 ± 0.03	1.60 ± 0.10
	7	<0.02	0.08 ± 0.01	0.92 ± 0.19	6.86 ± 0.10	17.67 ± 0.28	19.43 ± 0.21	97.13 ± 1.53	5.05 ± 0.34	2.13 ± 0.03	2.16 ± 0.02	1.47 ± 0.06
	14	<0.02	0.15 ± 0.03	1.08 ± 0.06	6.74 ± 0.03	17.5 ± 0.33	19.13 ± 0.21	133.67 ± 0.58	5.73 ± 0.48	2.79 ± 0.01	2.77 ± 0.01	1.27 ± 0.06
	21	0.02 ± 0.00	0.15 ± 0.02	1.33 ± 0.18	6.85 ± 0.01	20.00 ± 0.00	21.13 ± 0.12	157.33 ± 2.08	4.32 ± 0.23	2.65 ± 0.02	2.66 ± 0.03	1.40 ± 0.00
	28	0.02 ± 0.00	0.11 ± 0.03	0.92 ± 0.18	6.87 ± 0.01	21.67 ± 0.22	22.97 ± 0.06	90.00 ± 0.40	2.83 ± 0.03	2.45 ± 0.03	2.38 ± 0.02	1.33 ± 0.06
Global average		0.07 ± 0.12	0.14 ± 0.09	1.17 ± 0.33	6.85 ± 0.16	18.42 ± 1.72	20.18 ± 1.76	118.43 ± 27.51	5.51 ± 2.54	2.48 ± 0.25	2.44 ± 0.26	1.41 ± 0.13
Control phosphate bioreactor	0	0.35 ± 0.02	0.47 ± 0.01	0.67 ± 0.52	7.17 ± 0.01	17.33 ± 0.10	15.73 ± 0.15	112.00 ± 3.61	9.32 ± 0.65	1.22 ± 0.00	1.16 ± 0.02	1.53 ± 0.06
	7	<0.02	0.08 ± 0.02	1.01 ± 0.03	6.92 ± 0.09	17.67 ± 0.28	19.70 ± 0.00	131.00 ± 0.00	4.12 ± 0.01	1.12 ± 0.00	1.14 ± 0.00	1.57 ± 0.06
	14	<0.02	0.14 ± 0.00	0.97 ± 0.26	6.81 ± 0.02	17.5 ± 0.33	19.37 ± 0.06	153.33 ± 5.77	3.85 ± 0.27	1.11 ± 0.03	1.12 ± 0.01	1.40 ± 0.00
	21	0.02 ± 0.00	0.15 ± 0.01	3.07 ± 0.06	6.92 ± 0.02	20.00 ± 0.00	21.30 ± 0.00	421.00 ± 3.61	10.01 ± 0.25	0.99 ± 0.01	1.04 ± 0.02	1.47 ± 0.06
	28	0.03 ± 0.01	0.13 ± 0.01	3.08 ± 0.25	6.90 ± 0.01	21.67 ± 0.22	23.40 ± 0.00	378.00 ± 3.61	11.47 ± 0.15	1.06 ± 0.03	1.11 ± 0.00	1.40 ± 0.00
Global average		0.08 ± 0.11	0.14 ± 0.09	1.57 ± 0.94	6.97 ± 0.19	18.42 ± 1.72	20.66 ± 1.80	239.07 ± 147.97	7.75 ± 3.53	1.09 ± 0.09	1.11 ± 0.05	1.47 ± 0.08
Low phosphate bioreactor	0	<0.02	0.05 ± 0.00	2.55 ± 0.37	6.39 ± 0.03	17.33 ± 0.10	18.80 ± 0.26	283.00 ± 2.00	37.30 ± 0.44	0.06 ± 0.00	0.06 ± 0.00	0.90 ± 0.00
	7	<0.02	0.05 ± 0.00	2.81 ± 0.36	6.84 ± 0.10	17.67 ± 0.28	19.90 ± 0.00	275.00 ± 1.00	20.97 ± 0.50	0.09 ± 0.01	0.11 ± 0.00	1.20 ± 0.00
	14	<0.02	0.09 ± 0.01	2.69 ± 0.21	6.41 ± 0.01	17.5 ± 0.33	19.93 ± 0.06	395.67 ± 23.86	20.07 ± 0.32	<0.046	0.02 ± 0.00	0.80 ± 0.00
	21	0.02 ± 0.00	0.09 ± 0.00	2.66 ± 0.12	6.52 ± 0.03	20.00 ± 0.00	21.57 ± 0.06	406.67 ± 1.53	46.97 ± 0.83	<0.046	0.03 ± 0.00	0.83 ± 0.06
	28	0.02 ± 0.00	0.07 ± 0.00	2.60 ± 0.06	6.53 ± 0.01	21.67 ± 0.22	23.50 ± 0.00	312.33 ± 3.21	22.90 ± 0.36	<0.046	0.04 ± 0.00	1.00 ± 0.00
Global average		0.03 ± 0.01	0.08 ± 0.03	3.70 ± 1.76	6.66 ± 0.21	18.42 ± 1.72	21.18 ± 1.43	334.53 ± 62.52	29.64 ± 11.95	0.03 ± 0.09	0.05 ± 0.03	0.95 ± 0.16
Local drinking water	0	0.42 ± 0.01	0.42 ± 0.01	0.28 ± 0.32	7.00 ± 0.09	17.33 ± 0.10	15.5 ± 0.01	4.82 ± 1.17	0.17 ± 0.021	1.18 ± 0.02	1.16 ± 0.01	1.67 ± 0.06
	7	0.47 ± 0.03	0.70 ± 0.06	0.13 ± 0.13	7.10 ± 0.03	17.67 ± 0.28	13.67 ± 0.12	9.46 ± 0.96	<0.02	1.17 ± 0.01	1.18 ± 0. 02	1.53 ± 0.06
	14	0.33 ± 0.02	0.80 ± 0.05	0.29 ± 0.27	6.89 ± 0.04	17.5 ± 0.33	16.76 ± 0.12	32.47 ± 18.33	0.10 ± 0.01	1.21 ± 0.01	1.21 ± 0.010	1.37 ± 0.06
	21	0.38 ± 0.03	0.67 ± 0.08	0.40 ± 0.40	6.97 ± 0.01	20.00 ± 0.00	18.27 ± 0.15	11.63 ± 0.47	0.09 ± 0.01	0.83 ± 0.01	0.82 ± 0.011	1.37 ± 0.06
	28	0.43 ± 0.01	0.67 ± 0.02	0.21 ± 0.07	6.98 ± 0.04	21.67 ± 0.22	20.17 ± 0.12	3.96 ± 0.98	0.05 ± 0.01	1.23 ± 0.02	1.20 ± 0.01	1.37 ± 0.06
Global average		0.43 ± 0.07	0.72 ± 0.14	0.26 ± 0.22	7.14 ± 0.20	18.42 ± 1.72	16.61 ± 29	12.47 ± 11.63	0.10 ± 0.05	1.12 ± 0.17	1.11 ± 0.17	1.46 ± 0.14

It is shown the physicochemical parameters measured during 28 days of the DWBR and the local drinking water. The table indicates the measure taken every 7 days, with the average of the three replicas and the corresponding standard deviation.

### Microbial community structure

**Fig. 2:**
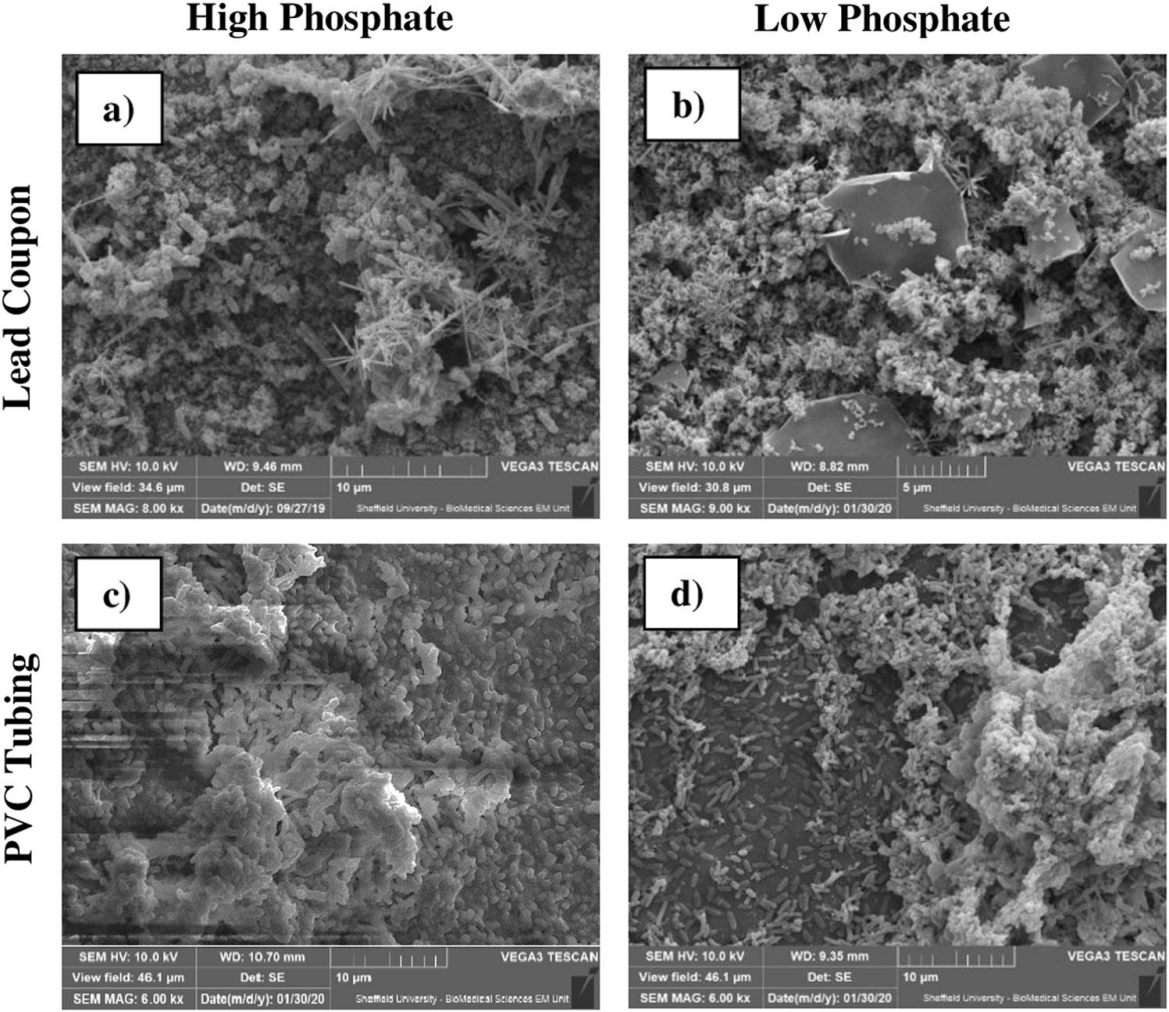
Scanning electron microscopy micrographs. Shown are scanning electron microscopy micrographs of lead coupons (**a**, **b**) and PVC tubing (**c**, **d**) for the experimental cases of high phosphate (**a**, **c**) and low phosphate (**b**, **d**). The scale is indicated as line with tick marks in the lower right corner of the figure, corresponding with 10µm (**a**–**d**) and 5µm (**b**).

Scanning electron microscopy (SEM) micrographs were performed to study the structure of biofilms developed over the surface of the materials used to build the bioreactors. Figure 2 shows the lead coupon surface and the section of PVC tubing under high and low phosphate experimental conditions. It is possible to observe a high accumulation of inorganic materials on the lead coupons, which made the visualization of microorganisms within biofilms difficult. However, individual microbial cells were observed for biofilms under high phosphate dosing. For PVC samples, individual microbial cells and biofilm-like structures were observed over all the analysed surface, especially in the case of low phosphate.

**Fig. 3:**
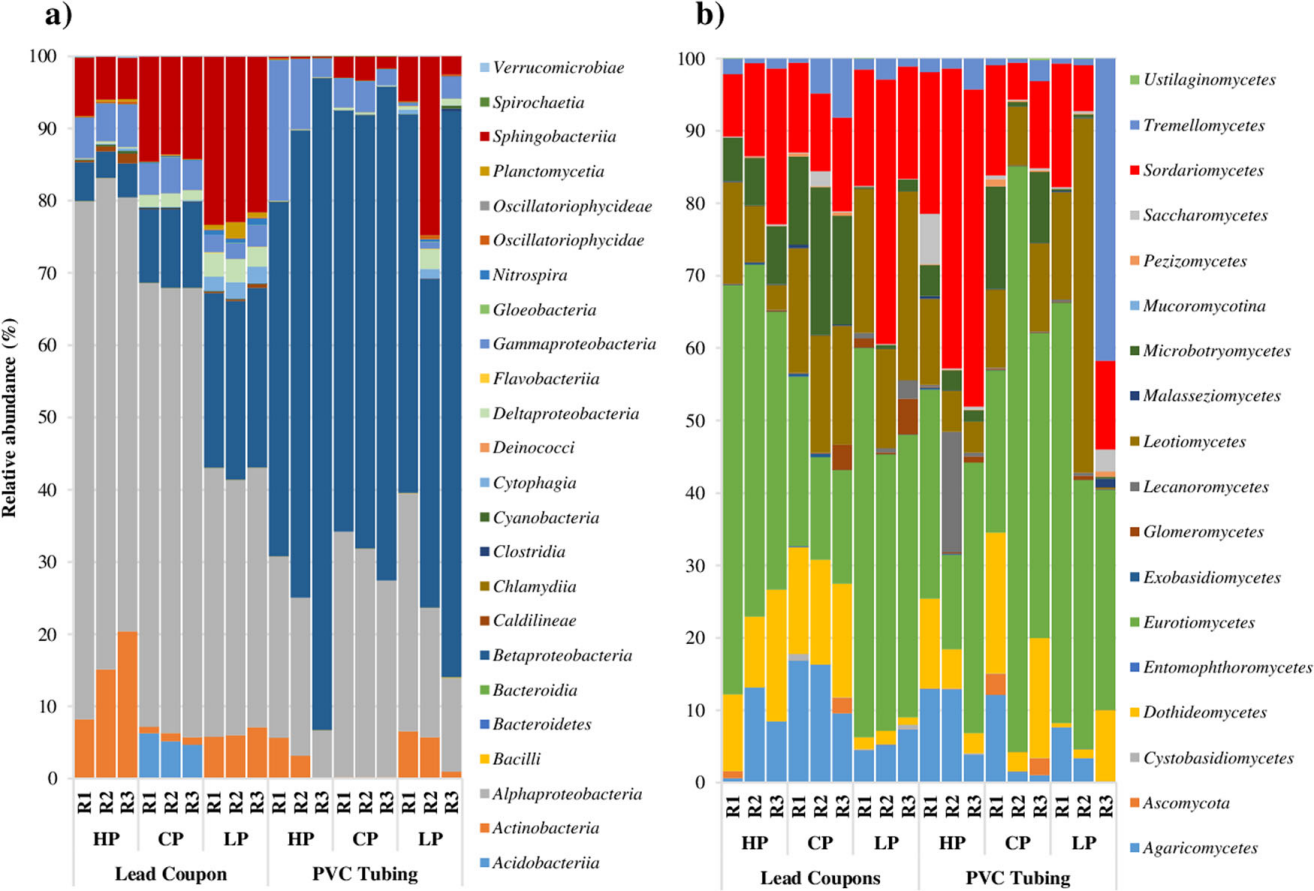
Bacterial and fungal classes. It is shown the relative abundance of the most abundant OTUs at class level of **a** bacteria and **b** fungi according to the treatment given (HP: high phosphate, CP: control phosphate and LP: low phosphate) and the material where the sample was taken (lead coupons and PVC tubing). R1, R2 and R3 are the replicas of each experimental case. Each replica is a pool of DNA extracted from four lead coupons and a piece of tubing of 75 cm.

Results of bacterial classes (Fig. 3a) showed that *Alphaproteobacteria* was the most abundant class in lead coupons (35–72%), but its abundance decrease in PVC tubing samples (6–34%), where *Betaproteobacteria* was more abundant (45–90%). Other classes with representative abundance were: *Actinobacteria* (0–20%) *Gammaproteobacteria* (0–19%) and *Sphingobacteria* (0–24%). Regarding fungal classes (Fig. 3b), sequences related to *Eurotiomycetes* (13–80%) and *Sordariomycetes* (5–44%) were detected with a considerably relative abundance in all the samples. Other classes that were highly represented in all the samples analysed included *Agaricomycetes* (0–17%), *Dothideomycetes* (1–19%), *Leotiomycetes* (8–49%), and *Microbotryomycetes* (0–20%).

**Fig. 4:**
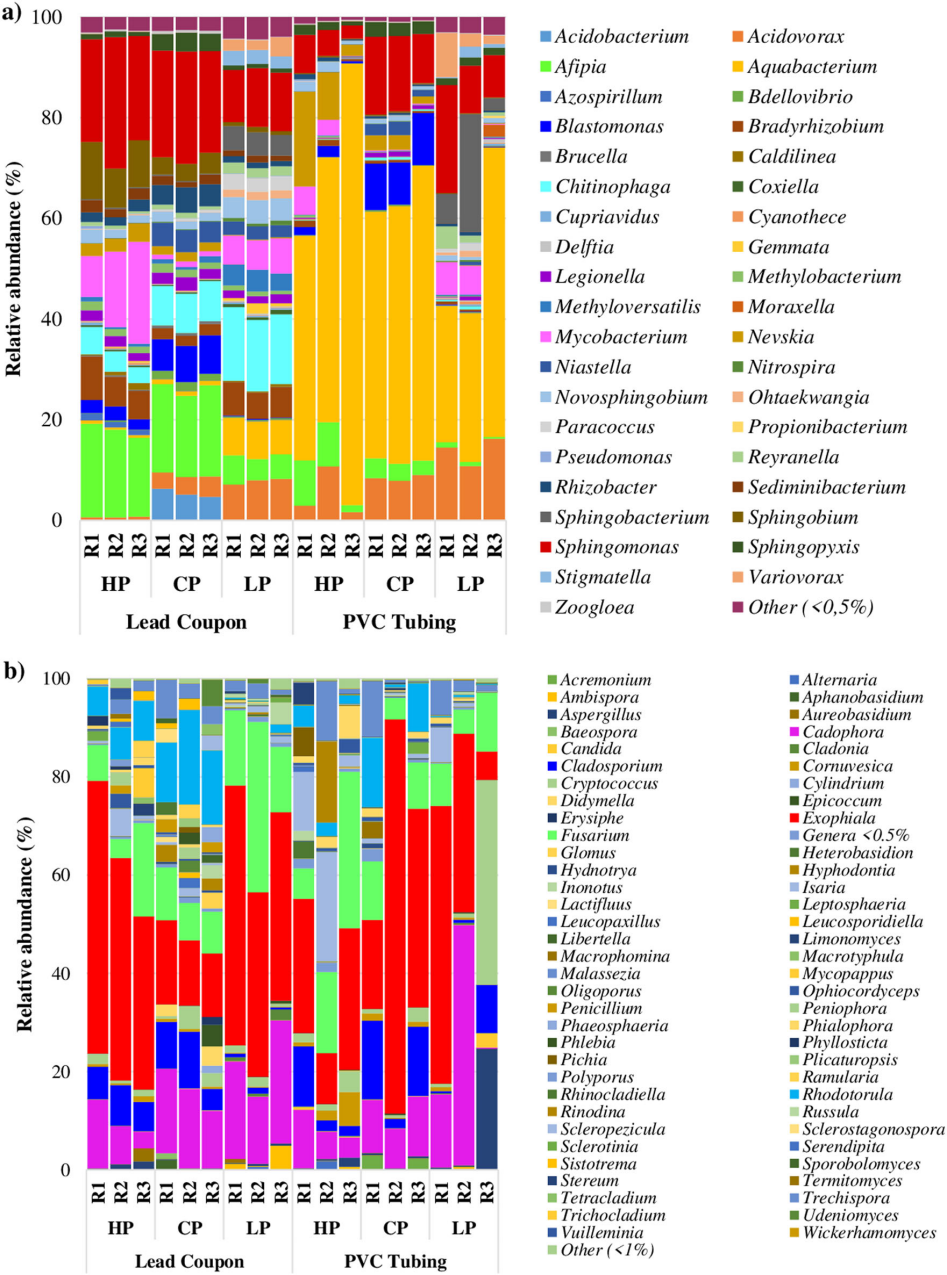
Bacterial and fungal genera. It is shown the relative abundance of the most abundant OTUs at genus level of **a** bacteria and **b** fungi according to the treatment given (HP high phosphate, CP control phosphate and LP low phosphate) and the material where the sample was taken (lead coupons and PVC tubing). R1, R2 and R3 are the replicas of each experimental case. Each replica is a pool of DNA extracted from four lead coupons and a piece of tubing of 75 cm.

Figure 4 shows the results for the relative abundance of the most abundant OTUs at genus level. The bacterial analysis (Fig. 4a) showed that the most abundant genera for lead coupon samples developed under the control phosphate case study were *Sphingomonas* (20–22%), *Afipia* (16–18%), *Chitinophaga* (8%), *Blastomonas* (6–8%) and *Acidobacterium* (5–6%). In the PVC tubing samples, under control phosphate condition, *Sphingomonas* and *Blastomonas* maintained a similar relative abundance when compared with lead coupons samples (10–15% and 8–10%) *Afipia* and *Acidobacterium* abundance was reduced (1–9% and 0%), while other genera increased their relative abundance like *Aquabacterium* (from 1 to 49–59%) and *Acidovorax* (from 3–4% to 8–9%).

The relative abundances of *Sphingomonas* and *Afipia* in lead coupons developed under high phosphate were similar when compared with control phosphate lead coupons samples (20–26% and 16–19%, respectively). However, other genera increased in abundance in phosphate-enriched samples like *Mycobacterium* (from 1 to 8–20%), *Sphingobium* (from 3–4% to 8–11%) or *Bradyrhizobium* (from 2 to 6–8%), while others reduced it like *Chitinophaga* (3–5%), *Blastomonas* (2–3%), *Acidobacterium* (0%) and *Acidovorax* (0%). However, in PVC tubing samples under high phosphate treatment, the relative abundance of the most abundant genera in lead coupons was reduced as follows: *Sphingomonas* (3–8%), *Afipia* (1–9%), *Mycobacterium* (0–6%), *Sphingobium* (0%) and *Bradyrhizobium* (0–1%). While, the abundance of other genera increased and this was particularly notable for *Aquabacterium* (45–88%), *Nevskia* (1–19%) and *Acidovorax* (2–11%).

Under low phosphate condition, in lead coupons samples genera like *Chitinophaga*, *Acidovorax*, *Aquabacterium, Sphingobacterium*, and *Variovorax* increased their relative abundance when compared with control and high phosphate samples (14–15%, 7–8%, 7%, 4–5% and 2–4%, respectively). Several genera such as *Sphingomonas* (10–12%), *Afipia* (4–6%), *Blastomonas* (0%), and *Sphingobium* (0%) decreased while other remained relatively well represented such as *Mycobacterium* (6–7%) and *Bradyrhizobium* (5–7%). Biofilm PVC samples developed under low phosphate treatment, had high relative abundance of *Aquabacterium* (27–58%), *Acidovorax* (11–16%), *Sphingomonas* (8–21%), *Sphingobacterium* (2–23%), *Variovorax* (2–9%) and *Mycobacterium* (0–6%).

In all the analysed samples, potential pathogenic genus such as *Legionella* were presented in biofilms form both type of materials, but with low relative abundance (0–3%), especially in lead coupons samples (1–3%).

When fungal relative abundance was analysed (Fig. 4b), the most represented genera in all the samples were *Cadophora* (0–49%), *Exophiala* (6–80%) and *Fusarium* (4–35%). *Cladosporium*, *Isaria* and *Rhodotorula* were more abundant in biofilm samples developed under phosphate-enriched treatment and in both type of materials (2–16%, 1–22% and 1–19%). *Cryptococcus* and *Aspergillus* were more abundant under low phosphate in both materials (1–42% and 0–25%). It is notable that *Candida*, a potential pathogenic fungus had very low relative abundance in the lead coupons samples (~0%), but its presence increased in PVC tubing samples, especially under low phosphate condition (0–3%). Please note that the replica 3 of PVC in the low phosphate case, had a considerable different composition when compared with the other replicas of the same case study.

### Venn diagrams of microbial community structure

**Fig. 5:**
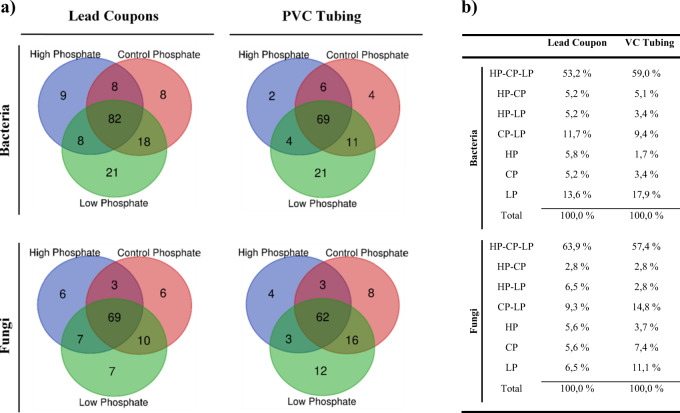
Venn diagrams of the microbial communities according to the treatment. **a** The figure shows Venn diagrams using the genera data of bacterial and fungal communities and classified according to the treatment of the samples (high, control and low phosphate) in the different experiments. **b** Percentage of the number of genera of bacteria and fungi of each Venn diagram treatment comparisons. HP high phosphate, CP control phosphate, LP low phosphate.

The Venn diagrams (Fig. 5a) show that independently of the treatment and material analysed, more than 50% of the bacterial genera were common to all the samples (Fig. 5b). What we consider as “unique” or “rare” bacterial genera, represented <10% for high phosphate and control phosphate samples, and between 6 and 18% for biofilm samples grown under low phosphate treatment, suggesting that the absence of PO_4_^3−^ might promote a higher abundance of unique or rare bacteria genera.

For fungi, the common genera for all samples consisted of 57–70% of the total relative abundance of all samples (Fig. 5b). The unique/rare genera were usually below a relative abundance of 10% in all the cases, except in the PVC tubing samples under low phosphate treatment (11.1%). Overall, these unique genera have a very low relative abundance for both type of microorganisms (bacteria and fungi) in all the samples.

### Non-metric multidimensional scaling analysis based on the relative abundance of microorganisms

**Fig. 6:**
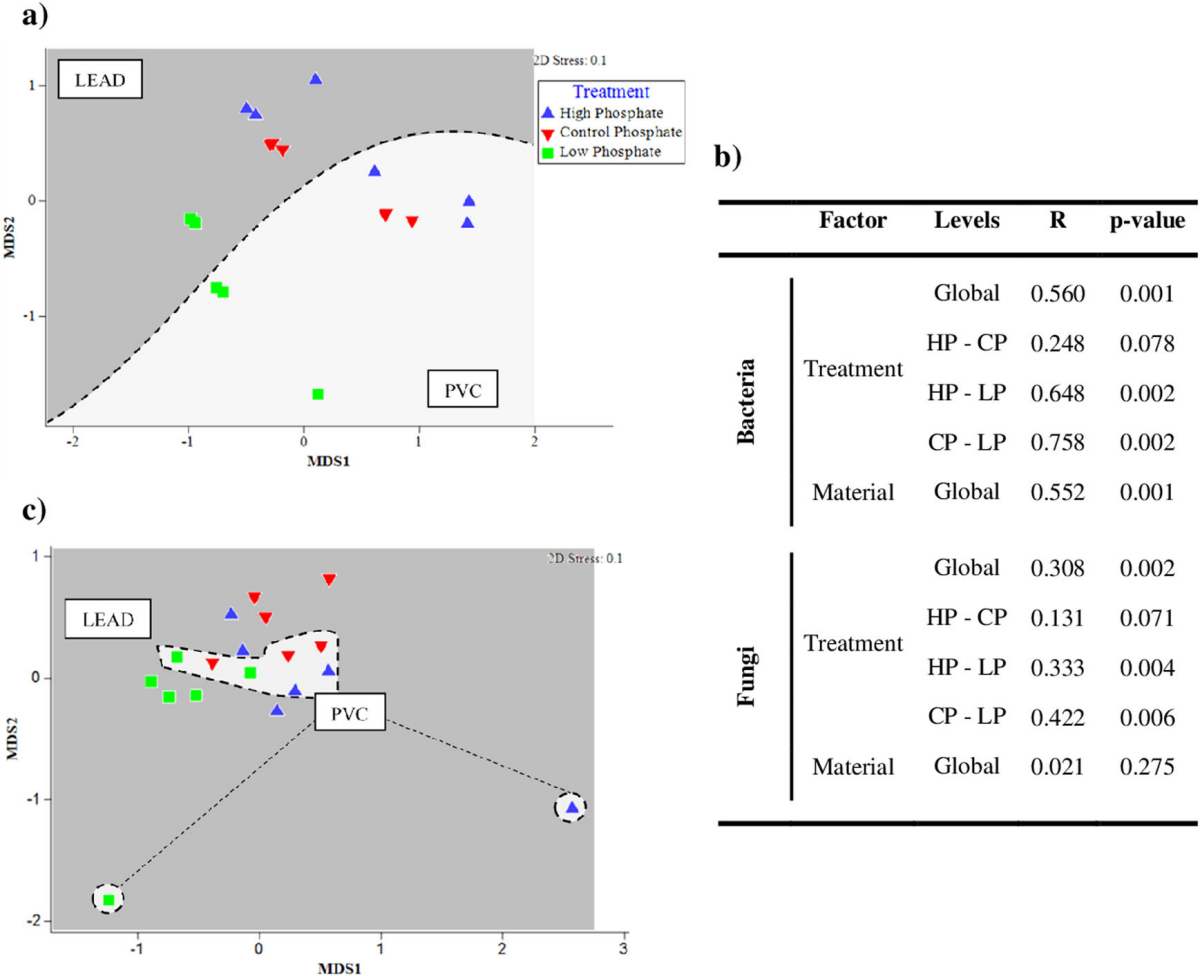
Non-metric multidimensional scaling and ANOSIM analysis. MDS plot based on the **a** bacterial and **c** fungal relative abundance of lead coupons and PVC tubing samples. The analysis was based on Bray–Curtis similarity matrix calculated from the relative abundance of bacteria OTUs at 97% cut off. The different groups are clustering according to the treatment used (high phosphate, control phosphate and low phosphate), a striped line separates both kind of materials (*n* = 18). **b** ANOSIM analysis. HP high phosphate, CP control phosphate, LP low phosphate.

Non-metric multidimensional scaling (MDS) analysis of bacteria at genera level, shows a clear separation of the bacterial community structure depending on both the PO_4_^3−^ treatment and the material used to support microbial growth (Fig. 6a). According to the Global ANOSIM analysis, considering all the samples (Fig. 6b), there were significant differences between the PO_4_^3−^ treatments (ANOSIM: Global-*R* = 0.560 *p*-value = 0.001), and between the materials (lead and PVC) (ANOSIM: Global-*R* = 0.552 *p*-value = 0.001). When treatments were compared to each other (pairwise comparisons), significant differences were found for the samples with PO_4_^3−^ presence (high and control phosphate) and the limited PO_4_^3−^ treatment (low phosphate) (ANOSIM high–low phosphate: *R* = 0.648 *p*-value = 0.002 and ANOSIM control–low phosphate: *R* = 0.758 *p*-value = 0.002, respectively).

The MDS analysis of fungal communities (Fig. 6c) did not show a clear separation between samples regarding PO_4_^3−^ treatments. The ANOSIM analysis indicated significant differences between the case studies when the factor PO_4_^3−^ treatment was considered (ANOSIM: Global-*R* = 0.308 *p*-value = 0.002). As observed for bacteria, significant differences were observed between the presence and absence of phosphate (ANOSIM HP-LP: *R* = 0.333 *p*-value = 0.004 and ANOSIM CP-LP: *R* = 0.422 *p*-value = 0.006). No significant differences were found associate to the type of material analysed (ANOSIM: Global-*R* = 0.021 *p*-value = 0.207).

### Alpha diversity

**Fig. 7:**
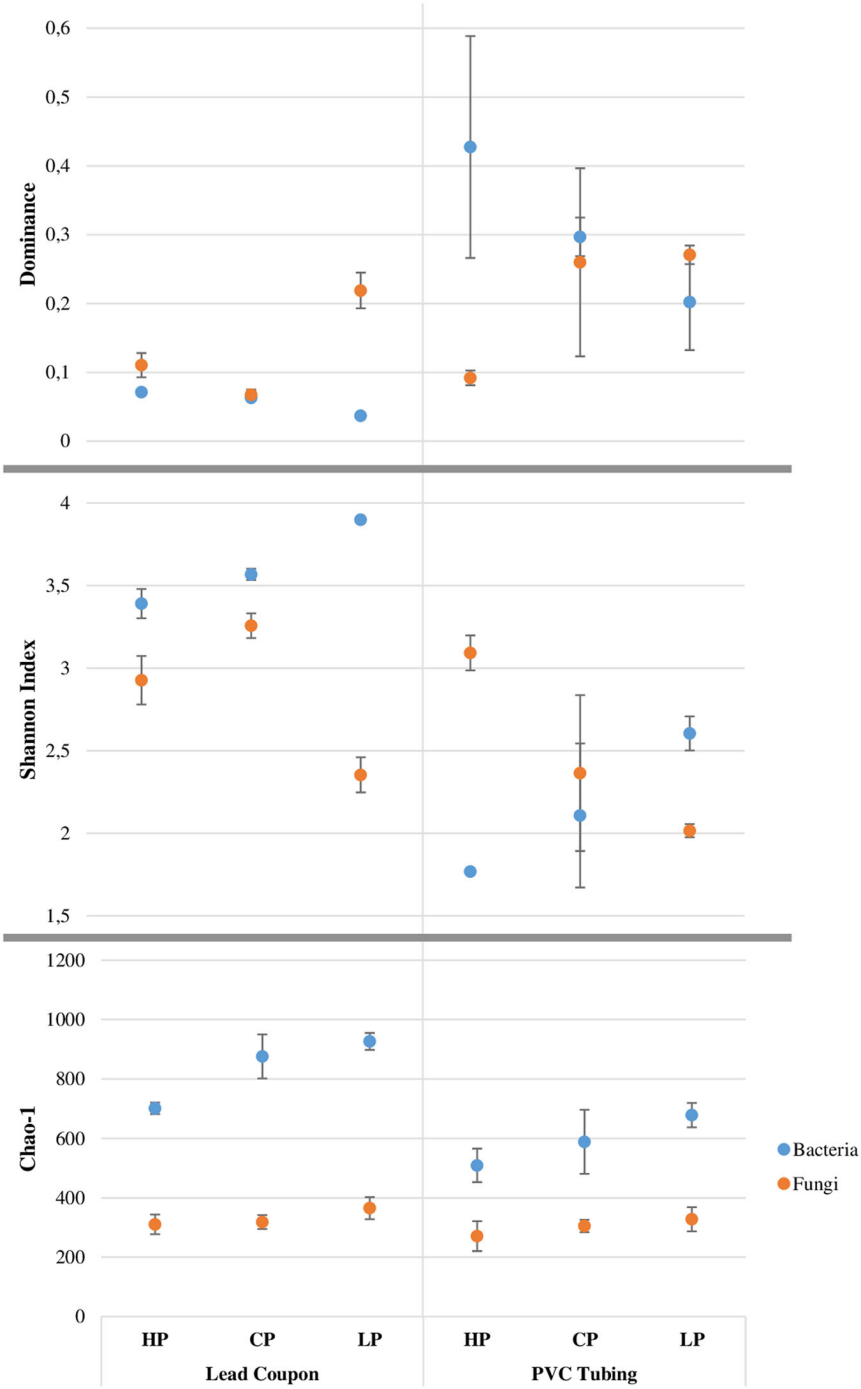
Dominance, Shannon (diversity index) and Chao I (richness index) for biofilm OTUs at 97% cut off. It is shown diversity indices average with standard errors (*n* = 3) for bacteria (blue markers) and fungi (orange markers) under different phosphate treatments (high phosphate, control phosphate and low phosphate) and different materials (lead coupon and PVC tubing).

Figure 7 shows the results of the alpha-diversity analysis; dominance, Shannon index (diversity) and Chao-1 (richness) and the results of the non-parametric test Mann–Whitney U (Supplementary Table 1) between samples. These showed that there were significant differences (*p*-value ≤ 0.05) between all the indices in samples from every experimental treatment on biofilms developed on lead coupons. However, no significant differences were observed for PVC tubing samples (*p*-value > 0.05). Furthermore, there were significant differences (*p*-value ≤ 0.05) between the indices according to the materials studied (lead and PVC).

Considering fungal communities, there were no significant differences (*p*-value > 0.05) in richness for all the samples and treatments analysed. There were significant differences (*p*-value ≤ 0.05) in dominance and diversity indices between treatments in lead coupons samples, and between high phosphate and low phosphate treatments in PVC tubing samples. However, when the indices were compared according to type material, only significant differences (*p*-value ≤ 0.05) were observed for dominance and diversity for biofilm samples under control phosphate treatment. When bacterial and fungal diversity indices were compared, there were significant differences (*p*-value ≤ 0.05) in all of cases, except in low phosphate PVC tubing samples (*p*-value > 0.05).

### Correlations between physicochemical and microbiological parameters

Supplementary Table 2 shows a matrix of the non-parametric Spearman’s correlations (two-tailed) between physicochemical and microbiological data (diversity indices and key representative genera) and indicates statistically significant correlations at different *p*-levels.

Regarding physicochemical parameters, all were correlated significantly (*p*-value < 0.05) between them. Water temperature was positively correlated with turbidity, iron and lead. However, it was negatively correlated with chlorine, orthophosphate, pH and TOC. Orthophosphate was positively correlated with chlorine, pH, TOC, but negatively with turbidity and iron, lead.

With reference to diversity indices, bacterial indices did not have significant correlations (*p*-value > 0.05) with physicochemical parameters, and it was noticed that bacterial diversity and richness were positively significant correlated (*p*-value < 0.05) with several genera like *Legionella*, *Mycobacterium* or *Sphingomonas*. Regarding fungal indices, diversity and dominance were significant correlated (*p*-value < 0.05) with all the physicochemical parameters and some genera like *Acidovorax*, *Afipia*, *Exophiala* and *Rhodotorula*. The correlations indicated that chlorine, pH, PO_4_^3−^ and TOC were positively significant correlated (*p*-value < 0.05) with fungal diversity. However, lead, iron, and turbidity were positively significant correlated (*p*-value < 0.05) with fungal dominance.

In relation to microbial genera (bacteria and fungi), three genera were strongly correlated with all the physicochemical parameters: *Afipia*, *Rhodotorula* and *Acidovorax*. *Aphia* and *Rhodotorula* were positively correlated with PO_4_^3−^, TOC and pH but negatively with lead, iron and turbidity, and the opposite was observed for *Acidovorax*.

## Discussion

Water companies invest considerable resources in developing effective and cost-effective methods to remove PO_4_^3−^ from wastewater effluents before is discharged back to the environment^26,27^ to prevent eutrophication events^31^. However, reducing PO_4_^3−^ in drinking water successfully, to promote the supply of drinking water free of or with less chemicals, was one of the biggest challenges of this study. This was specially challenged without significantly modifying any other characteristics that could affect the overall quality and safety of the drinking water supplied.

In this study, a concentration of 1.7 g/L of GEH104 iron pellets removed 96.08 ± 2.48% of PO_4_^3−^ from 1 L of drinking water (1.3 mg/L of PO_4_^3−^ as P) in 8 h. In other studies, removal of PO_4_^3−^ was also successful. For example, Genz et al.^28^ showed that granulated ferric hydroxide had a higher adsorption for PO_4_^3−^ and a higher affinity at low concentrations compared to other adsorbents like activated aluminium oxide, in assays with membrane bioreactors. Ernst et al.^32^ showed that in membrane bioreactors, a fixed-bed made of granular ferric hydroxide was able to maintain a total phosphorus concentration of <0.03 mg/L for municipal water reuse. Yousefi et al.^29^ reported that a dosage of 4.03 g of granular ferric hydroxide could remove 92.14% of PO_4_^3−^ in 82 min from an aqueous solution with an initial concentration of PO_4_^3−^ of 1.78 mg/L and pH = 5.81. Comparing with the other studies in wastewater systems mentioned before, the iron pellets tested in this study required more time to remove a comparable percentage of PO_4_^3−^, but the amount of pellet used was less. Therefore, the results in this paper ratified that iron pellets, in particular GEH104, can be used to successfully reduce the content of PO_4_^3−^ in drinking water without increasing significantly other key quality parameters, including turbidity.

In lead coupons, SEM micrographs (Fig. 2) show that biofilm-like structures were mixed with different chemical compounds which can form geometrical structures. Nadagouda et al.^33^, studying the formation of compounds on lead pipes in a laboratory-based DWDS, observed that pH values between 6 and 7, favoured the formation of chemical structures with different shapes, such as nanorods, microrods, and dendritic structures on lead surfaces with PO_4_^3−^ concentrations of at 3 mg/L. We were able to observe similar dendritic structures on the material surfaces (PVC and lead) analysed in this study. The composition of these structures was not analysed, but, according to other studies they might be apatite species. Peters et al.^34^, using spectrometric techniques to study lead service drinking water pipes, showed that lead carbonates were formed under pH adjusted-waters, and species like apatite were observed when orthophosphoric acid was added. This is in agreement with Hopwood et al.^35^, who found that lead water pipes from Yorkshire (UK), fed with PO_4_^3−^ treated drinking water, contained a variety of apatite minerals, included lead apatite species which are the least soluble form of lead^36^. In addition, microorganisms have the potential to induce metal precipitation in a range of environmental settings. Couasnon et al.^37^ tested the capacity of *Shewanella oneidensis* MR-1 biofilms to bio-induced the precipitation of lead-bearing mineral in aqueous cultures. The authors reported that pyromorphite was the first solid to precipitate within hours and that pyromorphite covered the surface of biofilms forming a crust. The appearance of these compounds was attributed to EPS and PO_4_^3−^ groups released by metabolically active cells in biofilms.

The results from the water physicochemical analysis showed that higher PO_4_^3−^ concentration favoured less lead and iron dissolution, thus proving the efficiency of PO_4_^3−^ as an anticorrosion agent in drinking water systems. PO_4_^3−^ has been reported to be a corrosion inhibitor that reduces metal content in water^5,38^. However, as observed in this study, PO_4_^3−^ addition can indirectly promote the increase of free chlorine in the water by reducing the amount of metal available to be oxidized by the disinfectant^39^. Chlorine decay in water supply systems can be attributed to the interaction between the pipe material, the attached biofilm and the accumulated sediment^40^. Chlorine can interact with organic matter in the pipes and favour the appearance of disinfection by-products like trihalomethanes (THM)^40,41^. It has been reported that bacteria isolated from drinking water supplies such as *Afipia* and *Methylobacterium* were able to degrade THM and haloacetic acids^42,43^. The presence of this type of bacteria could explain the reduction of free chlorine in low phosphate condition observed in this study. The results from this study agree with those of Jang et al.^21^, which reported an increase of chlorine in a reactor with corrosion-susceptible cast iron pipes in the presence of PO_4_^3−^. Increased free chlorine levels can reduce the number of active cells in the water thus indirectly controlling biofilm formation^44^. Despite this, chlorine has a limited effect on biofilms, due to the difficulty of penetrating the different biofilm layers^45^, especially if biofilms are thick, dense or with high EPS production^45^.

Several studies have reported that increasing PO_4_^3−^ levels in drinking water reduces the production of EPS in biofilms^22,46,47^. Among other functions, EPS is involved in protecting biofilm cells from external damage as well as maintaining its integrity and aiding the attachment of cells to substrates^48^. Reduction of EPS in response to PO_4_^3−^ increase can favour a weaker biofilm, sensitive to the action of oxidants like chlorine^45,49^ and more susceptible to its detachment from pipes. Xing et al.^49^, testing the effect of ozone-biologically activated carbon treatment with various PO_4_^3−^ doses in annular reactors, reported a decrease in the total amount of EPS, followed by a reduction in chlorine consumption and disinfection by-products formation. In agreement with these observations, this study showed that PO_4_^3−^ addition promoted a decrease in bacterial diversity in lead coupons.

We also observed a significant correlation between TOC and PO_4_^3−^. The influence of these two parameters as limiting nutrients able to control microbial growth has been widely discussed in the literature. In studies carried out in carbon-limited waters, the addition of phosphorus did not have any effect on microbial biofilm activity^24,50,51^. However, in drinking water with high amounts of organic matter, microbial growth can be limited by phosphorus instead of organic carbon^52–54^. Indeed, Park et al.^55^ suggested that TOC and PO_4_^3−^ interact having a combined impact on biofilm formation in drinking water systems. Butterfield et al.^56^ reported that iron oxide-containing corrosion products in drinking water promoted the adsorption of organic carbon and favoured biofilm growth. This could explain why in this study bacterial diversity was higher when PO_4_^3−^ and TOC were low but iron and lead content in the water were higher.

This study shows for the first time clear differences in the microbial community structure of mixed-species biofilm due to PO_4_^3−^ treatment. The addition of PO_4_^3−^ promoted the presence of microorganisms (e.g. *Sphingomonas*, *Bradyrhizobium*, and *Acidovorax*) related with mechanisms of phosphorus mobilization. For example, species belonging to the genera *Sphingomonas* can carry a gen involved in PO_4_^3−^ solubilization and transport^57^ like *gcd* gene^58^ and *pst* operon^59^. Similarly, *Bradyrhizobium* strains have the ability of solubilizing PO_4_^3−^ from hydroxyapatite^60^ in aqueous culture medium. Furthermore, the addition of PO_4_^3−^ in this study promoted the presence of microorganisms able to tolerate or accumulate heavy metals including *Acidovorax* and *Sphingomonas*^57,61^. We found fungi including *Aspergillus* and *Fusarium* that have been reported as important phosphorus solubilizing microorganisms^62–64^ in a range of environments. *Fusarium*, *Aspergillus*, *Exophiala* or *Rhodotorula* can tolerate and remove heavy metals from the environment^65–70^. For example, *Rhodotorula* when forming biofilms is successful at removing Pb^2+^ from aqueous solution thanks to the EPS production and the precipitation of lead with PO_4_^3−^ ^71^. The results from this study show that PO_4_^3−^ can promote specialised microorganisms in biofilms, both fungi and bacteria, which could have bioremediation applications, therefore promoting a protective environment inside the pipe.

Bacteria belonging to the genera *Sphingomonas*, *Afipia* and *Acidovorax*, and fungal genera such as *Exophiala*, *Cadophora* and *Fusarium*, were detected in all the conditions and materials studied. It can be suggested that these microorganisms form a core community and play a key role in biofilm formation, as proposed by other researchers studying mixed-species biofilms^72–77^. In these studies, bacteria belonging to the genera *Acinetobacter*, *Burkholderia*, *Methylobacterium*, *Mycobacterium*, *Sphingomonas* and *Staphylococcus* played an important role in the formation of biofilms an aggregates^78–81^.

When the microbial communities were analysed based on materials (lead vs. PVC), bacterial community structure and alpha-diversity indicators were significantly different. Samples from lead coupon samples were more diverse than PVC tubing samples. On the one hand, several studies in DWDS pipes made of different materials, agree that in plastic-based materials like PVC biofilm formation was lower when compared to cast iron^82^ or stainless steel^83^. On the other hand, other studies in drinking water systems support the opposite^17^, for example, Belcher^17^ reported that bacterial communities developed on PVC had a higher Shannon index than lead. In this study, lead was clearly promoting bacterial diversity and influenced the community structure of biofilms.

Fungal diversity was not significantly affected by type of material. This is in agreement with other studies where fungal communities in drinking water biofilms tended to be less diverse and more stable over time^73^ when compared with bacteria. From this study, we can confirm that fungi formed part of mix-species consortia with bacteria in biofilms, being an important component of the microbial communities of lead and PVC materials in DWDS^73,74^. Despite fungi are not usually included in drinking water regulations, the monitoring of these organisms should be considered by water utilities due to their involvement in processes that can deteriorate water quality.

The results from this study were performed in bioreactors and despite the dynamic conditions under which the biofilms were grown, further studies using real scale DWDS and hydraulic conditions like those in real water networks will help to reinforce findings from this research.

Future studies will benefit from considering the role of other physicochemical parameters at different PO_4_^3−^ concentrations in lead pipes, like manganese, nitrate or THM. Particularly, the monitoring of manganese could be of interest due to its ability to accelerate the oxidation of Pb(II) carbonate solids by free chlorine in lead pipes^84^. Another aspect of this study that could be developed further is the detection of specific pathogens and antibiotic resistance genes due to phosphate dosing in lead pipes. We found that lead promoted the appearance of pathogenic species but at very low relative abundance. Longer studies (> 28 days) will help to elucidate the role of biofilms as reservoirs of pathogens under phosphate enrichened conditions.

The results of this research show several key findings for the management of DWDS as follows:

- Iron pellets GEH104 were effective at reducing PO_4_^3−^ in drinking water, removing the 96.08 ± 2.48% of PO_4_^3−^ in 8 h.
- Phosphate dosing of 2 mg/L was successful at reducing metal content and lead dissolution in drinking water, comparing with the other conditions studied (below 1 and 1 mg/L).
- Phosphate dosing had a significantly stronger effect on bacterial than on fungal communities on both type of materials (lead and PVC).
- Lead favoured bacterial diversity, while phosphate reduced it and increased fungal diversity in biofilms.
- The combined presence of phosphate and lead materials favoured the appearance of microorganisms specialized in phosphorus metabolism and metal resistance. Some of these microorganisms have bioremediation capabilities, therefore can be used to promoting a protective environment inside the pipe.

The data from this study yields new information about the consequences of PO_4_^3−^ variation in DWDS. Showing that lead promoted a more diverse bacterial community than PVC and that phosphate reduced bacterial biofilm diversity in both type of materials, while increasing fungal diversity. Therefore, it can be concluded that phosphate variation can induce changes in the structure of biofilms and affect the level of chlorine in the water, impacting the disinfection strategies where a chlorine residual is kept safeguarding water safety. This new knowledge is of great relevance for water utilities to improve management strategies and inform decisions to control microbial growth in drinking water pipelines.

## Methods

### Bioreactor system set up

**Fig. 8:**
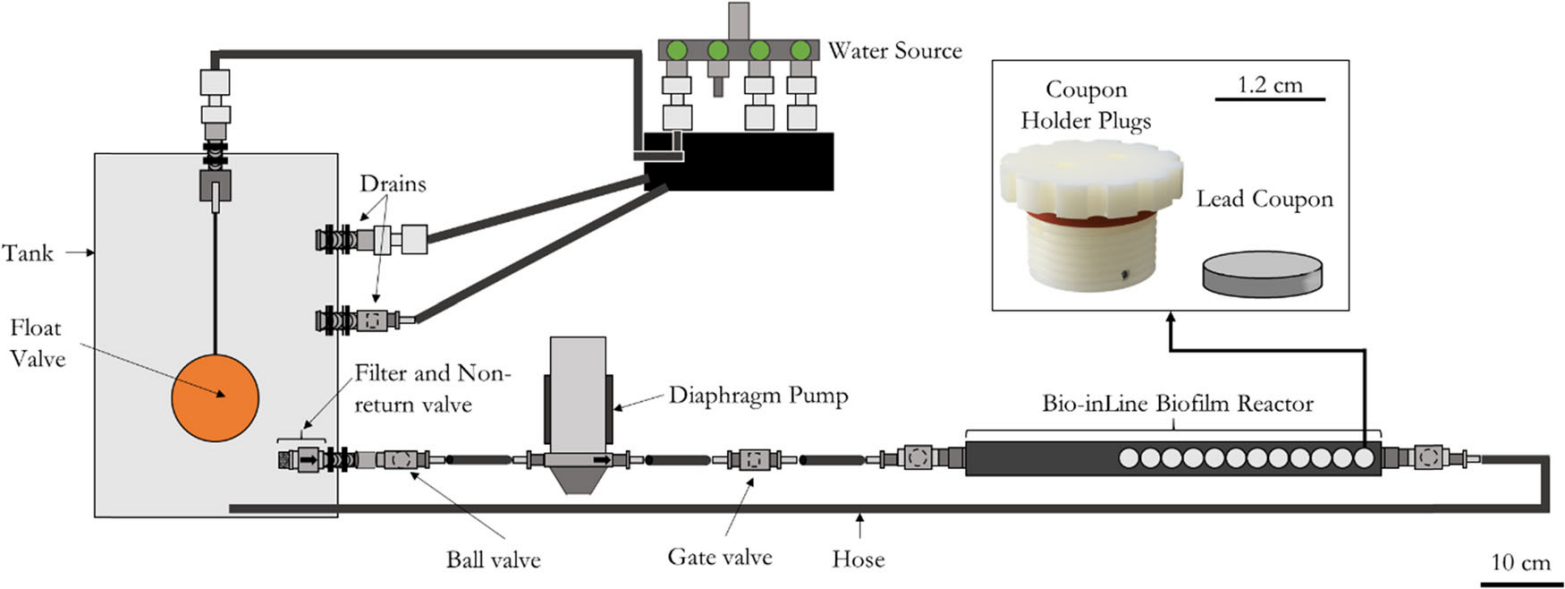
Schematic representation of the drinking water biofilm reactor (DWBR). The system is composed by a tank of 25 L connected to a source of local tap water, a float valve which controls the entrance of water to the tank, a diaphragm pump to create a constant flow (5.3 L/min), and one Bio-inline bioreactor with 12 lead coupons. Ball valves and gate valves were added to control the flow through the system, and two drains (one principal and one for emergency), were installed to renew the water inside the tank twice per day (flow of 35 mL/min). The scale is indicated as a line in the lower right corner of the figure, corresponding with 10 cm.

In order to study the development of microorganisms under different doses of phosphate, three drinking water biofilm reactors (DWBR) were designed and assembled for each experimental case (Fig. 8).

Each DWBR is composed by a tank of 25 L capacity with a lid connected to a source of local tap water, a float valve which controls the entrance of water to the tank, a high-pressure diaphragm pump (Flojet Series R3811/03811 XYLEM) to create a constant flow of 5.3 L/min, which was the flow recommended for lead pipes sampling^85^, and one Bio-inline bioreactor (Model IBR-500 Bio-inLine^®^ Biofil Reactor, Biosurface Technologies Corporations, Bozeman, USA) with 12 lead coupons (1.27 cm of diameter) (Heaps Arnold & Heaps LTD, Rotherham, UK). Ball valves and gate valves were added to control the flow through the system, and two drains (one principal and one secondary for emergency), were installed to renew the water inside the tank twice per day (flow of 35 mL/min). To connect all the parts of the bioreactor, a black PVC flexible tubing (inner diameter: 9.75 mm) (RS PRO, Northants, UK) was used. The water used for the experiments was the local drinking water supply, where the water is treated with ferric sulphate at treatment level (as coagulant) and monosodium phosphate is used to minimise lead dissolution during distribution. The target PO_4_^3−^ dose is 1.3 mg as P/L, but this varied during the experiment.

The DWBR were cleaned before starting the experiment with Rodolite H (RODOL Ltd, Liverpool, UK), a solution of sodium hypochlorite with less than 16% free chlorine, at a concentration of 20 mg/L^73^. Lead coupons were cleaned by submerging them for 45 min in a solution of RBS 2% (RBS^®^ 35 concentrate, Fluka Analytical), a general cleaning detergent composed by a mixture of anionic and non-ionic surfactants, and by shaking them at 100 rpm. Then, the coupons were cleaned during 15 min in distilled water at 100 rpm and, finally, they were sterilised with UV light, with an exposure time of 30 min^86^.

To feed the DWBR with high PO_4_^3−^, a concentration of 65 mg/L of sodium dihydrogenorthophosphate (monosodium phosphate 32%, Airedale Chemicals, Keighley, UK), was added to double the normal concentration of phosphate in the local drinking water and supplied with a peristaltic pump at a speed of 17 mL/min. For the DWBR with a PO_4_^3−^ limiting concentration, PO_4_^3−^ was removed with GEH104 iron pellets (GEH Wasserchemie GMBH & CO. KG, Osnabrück, Germany) at a concentration of 2 g/L. A pre-treatment tank containing the iron pellets used to remove the PO_4_^3−^ from the main drinking water supply was needed. In the pre-treatment tank, the pellets were located into filter bag shocks (REDSEA) overlapped with a hole size of 225 and 150 µm, and the pellets were changed three times per week. The pre-treatment tank was built in a higher elevated position than the tank connected to the DWBR (to favour the transport of water between the two tanks), and a diaphragm pump was added to ensure that all the water in the tank passed through the filter bag containing the iron pellets.

### Phosphate kinetic studies: removal of phosphate from the local drinking water supply

In order to remove PO_4_^3−^ from the local drinking water supply, a set of experiments were performed using two commercial iron pellets (granulated ferric hydroxide): GEH104 (GEH Wasserchemie GMBH & CO. KG, Osnabrück, Germany) and WARP (Aquaminerals B.V, Nieuwegein, Netherlands). WARP (Aquaminerals B.V, Nieuwegein, Netherlands) and GEH104 (GEH Wasserchemie GMBH & CO. KG, Osnabrück, Germany). WRAP are pellets of 1–4 mm, red/brown in colour, and they are predominantly formed of ferrihydrite (Fe_5_HO_8_·4H_2_O). GEH104 pellets have a size of 0.2–2 mm, black colour, and are composed mostly of akageneite (ß-FeOOH) and ferric oxyhydroxide (Fe(OH)_3_).

To prevent PO_4_^3−^ absorption on glass surfaces, note that for all the experiments involving iron pellets, all the glassware was washed with a 10% HCl solution for 30 min, rinsed with abundant distilled water and dried in a convection oven at 60 °C. Before starting the tests, the pellets were pre-treated by rising them (100 g) with ultrapure deionized water 10 times, then, the pellets were oven-dried at 105 °C overnight.

To compare the PO_4_^3−^ adsorption capacity of the iron pellets, three different quantities (0.2, 0.5 and 1 g) of each type of pellet was mixed in 1 L of local drinking water within a flocculator (Stuart Equipment SW6, Staffordshire, UK) at 150 rpm during 8 h at room temperature. To obtain an absorption kinetic curve of each pellet, the concentration of PO_4_^3−^ was measured every 2 h. A sample aliquot of 10 mL was taken from each experimental case and the PO_4_^3−^ concentration was measured using PhosVer® 3 Phosphate Reagent Powder Pillows (HACH) and a spectrophotometer set to a wavelength of 880 nm (Jenway 7300 Visible Spectrophotometer).

### Physicochemical analysis of water quality

During the 28 days of the experiment, several physicochemical factors were analysed daily for the water in the three DWBR as well as the local drinking water feeding them. Every analysis was performed in triplicate and the average of the replicates was calculated. Several parameters were measured daily in the laboratory; turbidity by means of a Palintest turbidity meter (Palintest PTH7091, UK), chlorine (free and total) with a chlorine meter (ChloroSense Palintest, UK), temperature and pH using a HannaH1991003 pH-meter (Hanna Instruments, Bedfordshire, UK) and orthophosphate concentration which was measured as explained above with PhosVer® using a Jenway 7300 Visible spectrophotometer. In addition, triplicate bulk water samples were collected weekly, and analysed for total lead, total iron, orthophosphate, total phosphorus and total organic carbon by an accredited drinking water laboratory, ALS environmental (Coatbridge, UK).

### Biofilm sampling from bioreactors

After 28 days of biofilm development, lead coupons were collected from the bioreactor and placed in a sterile petri dish with 30 ml of phosphate-buffered saline (PBS) (pH 7). Then coupons were brushed using toothbrushes to remove the biofilm attached to the coupon surface as described in Deines et al.^87^. The toothbrushes were previously sonicated in an Ultrasonic Water Bath (Model AL04-04-230, Advantage-Lab, Menen, Belgie) during 45 min in a solution of 2% of RBS and during 10 min in distilled water, and then, they were sterilized in an autoclave (Autoclave Prestige 2100 Classic 9 litres, Prestige Medical, Blackburn, UK) during 20 min at 121 °C. The biofilm solutions were filtered through a sterile filter (0.22 µm MCE Membrane MF-Millipore, UK), and the filters were kept at −20 °C until DNA extraction was performed.

In addition to the lead coupons, three pieces of PVC flexible tubing of 75 cm from each bioreactor were taken after 28 days to study biofilm growth on this material. A longitudinal cut along the tubes was performed with a sterile scalpel, and then, each half-tube was brushed internally using sterile toothbrushes and 30 mL of PBS (pH 7). The PBS solutions with the biofilms were filtered through a sterile filter (0.22 µm MCE Membrane MF-Millipore, UK), and filters kept at −20 °C until DNA extraction.

### Extraction of DNA

The procedure for extracting DNA was based on a modified protocol described by Douterelo et al.^12^. Filters with concentrated biofilm solution, were placed into a 2 mL Eppendorf tubes. Then 740 μl of SET lysis buffer (40 mM EDTA, 50 mM Tris–HCl, pH 9, 0.75 M sucrose) was added and the filters were crushed with sterilized pestles (autoclave 20 min 121 °C). Ninety microlitres of lysozyme (9 mg/ml) was added and filters incubated at 37 °C for 30 min with shaking (100 rpm) in a Hybaid hybridisation oven (Thermo Scientific, UK). Subsequently, 90 μl of sodium dodecyl sulphate (SDS) and 25 μl of proteinase K (20 mg/ml) were added to the tube and the sample incubated at 55 °C for 2 h with shaking in a Hybaid oven. The supernatant (aqueous phase) was transferred to a clean 2 mL Eppendorf tube, and then 137 μl 5 M NaCl and 115 μl CTAB/NaCl solution (100:41 mg/ml) were added and incubated at 65 °C for 30 min with shaking in a Hybaid oven. The supernatant was treated twice with 838 μl of chloroform:isoamyl alcohol (24:1) (Sigma, UK) and DNA was precipitated with 815 μl of isopropanol. The pellet was then washed twice in 1 ml of 70% ethanol, dried for 30 min and re-dissolved in 50 μl DEP-treated sterile water. Quantity and purity of the extracted DNA were assessed using NanoDrop ND-1000 spectrophotometer (Nanodrop, Wilmington, USA).

### Sequencing analysis

Sequencing was performed by Illumina MiSeq technology by MR DNA Molecular Research Laboratory (www.mrdnalab.com, Texas, USA) following the manufacturer’s guidelines. For studying the bacterial communities, the 16S rRNA gene was sequenced using primers 28F and 519R. For fungal characterisation, the ribosomal internal transcribed spacer regions ITS1-2 were targeted. Sequence data was processed by MR DNA using as described in Douterelo et al.^74^. The number of shared genera between samples and the Venn diagrams was calculated using the web tool provided by the Bioinformatics & Evolutionary Genomics group at the University of Gent (http://bioinformatics.psb.ugent.be/webtools/Venn/). Alpha-diversity metrics including dominance, Shannon Index (diversity, number of different OTUs taking into account their relative abundance) and Chao-1 (richness, number of different OTUs) of bacterial and fungal communities at 97% sequence similarity cut off, were calculated with the software PAST version 4.0^88^.

### Scanning electron microscopy

On day 28, a lead coupon and a piece of PVC tubing were taken from the high phosphate and low phosphate experimental bioreactors for visualization with SEM by the Electron Microscopy Facility of the Faculty of Science of the University of Sheffield. The coupons were fixed for 24 h in 2.5% glutaraldehyde (Glutaraldehyde, 25% aqueous solution, Thermo Fisher, UK) and washed in PBS and post fixed using 2% aqueous Osmium tetroxide, washed briefly in water and dehydrated. The dehydration consisted of a series of treatments with graded ethanol, samples were dried in a 50/50 mixture of 100% ethanol and hexamethyldisilazane (HEX) with a final drying step in 100% HEX. Samples were mounted onto a pin-stub using a Leit-C sticky tab and Leit-C plast, gold-coated using an Edwards S150B sputter coater and examined in using a Tescan Vega3 LMU SEM.

### Statistical analysis

To establish differences in microbial communities (bacteria and fungi), the sequences relative abundance at 97% similarity cut off was used. All data were transformed by square root calculations and Bray–Curtis similarity matrixes were generated using the software Primer-E v7 (PRIMER-E, Plymouth, UK)^89^ and visualised using non-metric multidimensional scaling (MDS) diagrams. The analysis of similarity statistics (ANOSIM) was calculated using the same Bray–Curtis similarity distance matrixes using Primer-E v7. R statistic values from the MDS analysis indicated the strength of the impact that the factors had on the samples^73^, in this case treatment (high phosphate, control phosphate, low phosphate) and material (lead and PVC). R values vary between 0 and 1, where 1 indicates high separation of the samples between levels of the factor and 0 indicates no separation^73^.

The statistical differences between alpha-diversity metrics in each sample were tested by performing a non-parametric Mann–Whitney U test with SPSS Statistics 26 (IBM, USA). Correlations between physicochemical and microbiological parameters were explored by Spearman’s rank non-parametric correlations using SPSS Statistics 26 (IBM, USA). Alpha-diversity metrics and the relative abundance of the most representative taxonomic genera for bacteria and fungi were used as microbiological parameters in the establishment of correlations.

### Reporting summary

Further information on research design is available in the Nature Research Reporting Summary linked to this article.

## Supplementary information

Supplementary Information

Reporting Summary

## Data Availability

Raw sequence data that support the findings of this study have been deposited in NCBI library as a Sequence Read Archive (SRA) with the accession code PRJNA663268 (https://www.ncbi.nlm.nih.gov/Traces/study/?acc=PRJNA663268).
