# Supplementary material for: Influence of phosphate dosing on biofilms development on lead in chlorinated drinking water bioreactors
Source: NPJ Biofilms Microbiomes. 2020 Oct 23;6:43. doi: 10.1038/s41522-020-00152-w (PMC7585443; doi:10.1038/s41522-020-00152-w)
Supplement: Supplementary file 1 — Supplementary Information [file 41522_2020_152_MOESM1_ESM.pdf]

SUPPLEMENTARY MATERIAL

Influence of phosphate dosing on biofilms development on lead in chlorinated drinking water bioreactors

Gonzalo Del Olmo[1], Arslan Ahmad[3,4,5], Henriette Jensen[2], Esther Karunakaran[2], Esther Rosales[1], Carolina Calero[1], Paul Gaskin[6], Isabel Douterelo[1*]

[1]Department of Civil and Structural Engineering, University of Sheffield, Sheffield, United Kingdom.

[2]Department of Chemical and Biological Engineering, University of Sheffield, Sheffield, United Kingdom.

[3]KWR Water Cycle Research Institute, Groningenhaven 7, 3433 PE Nieuwegein, The Netherlands.

[4]KTH-International Groundwater Arsenic Research Group, Department of Sustainable Development, Environmental Science and Engineering, KTH Royal Institute of Technology, Teknikringen 10B, SE-100 44 Stockholm, Sweden.

[5]Department of Environmental Technology, Wageningen University and Research (WUR), Droevendaalsesteeg 4, 6708 PB Wageningen, The Netherlands.

[6]Dwr Cymru Welsh Water, Newport, United Kingdom.

Supplementary Table 1: Statistical differences between Diversity Indices of each group based on non-parametric test Mann-Whitney U. *In green significant differences according to the p-value (≤ 0,05).*

	Material	Treatment	Dominance	Shannon Index	Chao-1
**Bacteria**	Lead	HP-CP	0.05	0.05	0.05
		HP-LP	0.05	0.046	0.05
		CP-LP	0.05	0.046	0.05
	PVC	HP-CP	0.827	0.827	0.827
		HP-LP	0.275	0.275	0.275
		CP-LP	0.275	0.75	0.275
	Lead-PVC	HP-HP	0.05	0.05	0.05
		CP-CP	0.05	0.05	0.05
		LP-LP	0.05	0.046	0.05
**Fungi**	Lead	HP-CP	0.046	0.05	0.827
		HP-LP	0.05	0.05	0.275
		CP-LP	0.046	0.05	0.275
	PVC	HP-CP	0.275	0.127	0.827
		HP-LP	0.05	0.05	0.658
		CP-LP	0.513	0.513	0.827
	Lead-PVC	HP-HP	0.827	0.513	0.513
		CP-CP	0.046	0.05	0.513
		LP-LP	0.127	0.05	0.275
**Bacteria and Fungi**	Lead	HP	0.05	0.05	0.127
		CP	0.05	0.05	0.05
		LP	0.05	0.05	0.05
	PVC	HP	0.05	0.05	0.05
		CP	0.046	0.05	0.05
		LP	0.513	0.513	0.127

Supplementary Table 2: Sperman´s rank non-parametric correlations of physicochemical and microbiological parameters.

	Material	Water Temperature	Total Chlorine	Free Chlorine	Turbidity	pH	Orthophosphate	Phosphorus	Iron	Lead	TOC
**Material**	1,000										
**Water Temperature**	0,000	1,000									
**Total Chlorine**	0,000	-.866**	1,000								
**Free Chlorine**	0,000	-.500*	.866**	1,000							
**Turbidity**	0,000	1.000**	-.866**	-.500*	1,000						
**pH**	0,000	-.500*	.866**	1.000**	-.500*	1,000					
**Orthophosphate**	0,000	-1.000**	.866**	.500*	-1.000**	.500*	1,000				
**Phosphorus**	0,000	-1.000**	.866**	.500*	-1.000**	.500*	1.000**	1,000			
**Iron**	0,000	1.000**	-.866**	-.500*	1.000**	-.500*	-1.000**	-1.000**	1,000		
**Lead**	0,000	1.000**	-.866**	-.500*	1.000**	-.500*	-1.000**	-1.000**	1.000**	1,000	
**TOC**	0,000	-.500*	.866**	1.000**	-.500*	1.000**	.500*	.500*	-.500*	-.500*	1,000
**Bacteria dominance**	.867**	-0,341	0,318	0,210	-0,341	0,210	0,341	0,341	-0,341	-0,341	0,210
**Bacteria diversity**	-.867**	0,341	-0,318	-0,210	0,341	-0,210	-0,341	-0,341	0,341	0,341	-0,210
**Bacteria richness**	-.696**	0,459	-0,341	-0,131	0,459	-0,131	-0,459	-0,459	0,459	0,459	-0,131
**Fungi dominance**	0,311	.590**	-.682**	-.590**	.590**	-.590**	-.590**	-.590**	.590**	.590**	-.590**
**Fungi diversity**	-0,332	-.616**	.681**	.564*	-.616**	.564*	.616**	.616**	-.616**	-.616**	.564*
**Fungi richness**	-0,268	0,262	-0,261	-0,190	0,262	-0,190	-0,262	-0,262	0,262	0,262	-0,190
**Acidovorax**	.568*	.695**	-.591**	-0,328	.695**	-0,328	-.695**	-.695**	.695**	.695**	-0,328
**Afipia**	-.739**	-.525*	.545*	0,420	-.525*	0,420	.525*	.525*	-.525*	-.525*	0,420
**Aquabacterium**	.867**	0,131	-0,091	-0,026	0,131	-0,026	-0,131	-0,131	0,131	0,131	-0,026
**Chitinophaga**	-.867**	0,315	-0,204	-0,039	0,315	-0,039	-0,315	-0,315	0,315	0,315	-0,039
**Mycobacterium**	-.568*	-0,013	-0,295	-.525*	-0,013	-.525*	0,013	0,013	-0,013	-0,013	-.525*
**Sphingomonas**	-.589*	-0,026	0,182	0,289	-0,026	0,289	0,026	0,026	-0,026	-0,026	0,289
**Aspergillus**	0,246	-0,184	-0,023	-0,223	-0,184	-0,223	0,184	0,184	-0,184	-0,184	-0,223
**Exophiala**	-0,075	0,131	-0,204	-0,223	0,131	-0,223	-0,131	-0,131	0,131	0,131	-0,223
**Fusarium**	-0,096	0,131	-0,250	-0,302	0,131	-0,302	-0,131	-0,131	0,131	0,131	-0,302
**Penicillium**	-0,118	-0,236	0,409	.472*	-0,236	.472*	0,236	0,236	-0,236	-0,236	.472*
**Rhodotorula**	-0,289	-.511*	.772**	.826**	-.511*	.826**	.511*	.511*	-.511*	-.511*	.826**

	Bacteria dominance	Bacteria diversity	Bacteria richness	Fungi dominance	Fungi diversity	Fungi richness	Acidovorax	Afipia	Aquabacterium	Chitinophaga	Mycobacterium	Sphingomonas	Aspergillus	Exophiala	Fusarium	Penicillium	Rhodotorula
**Material**																	
**Water Temperature**																	
**Total Chlorine**																	
**Free Chlorine**																	
**Turbidity**																	
**pH**																	
**Orthophosphate**																	
**Phosphorus**																	
**Iron**																	
**Lead**																	
**TOC**																	
**Bacteria dominance**	1,000																
**Bacteria diversity**	-.996**	1,000															
**Bacteria richness**	-.876**	.889**	1,000														
**Fungi dominance**	0,115	-0,107	-0,094	1,000													
**Fungi diversity**	-0,143	0,135	0,146	-.977**	1,000												
**Fungi richness**	-0,425	0,425	0,405	0,147	-0,170	1,000											
**Acidovorax**	0,263	-0,269	-0,158	.555*	-.612**	-0,027	1,000										
**Afipia**	-.538*	.534*	0,317	-.676**	.682**	0,187	-.734**	1,000									
**Aquabacterium**	.767**	-.759**	-.600**	0,341	-0,358	-0,256	.587*	-.767**	1,000								
**Chitinophaga**	-.926**	.924**	.843**	-0,103	0,141	0,448	-0,321	.515*	-.701**	1,000							
**Mycobacterium**	-.598**	.591**	0,383	0,061	-0,024	0,212	-0,280	0,383	-.736**	0,424	1,000						
**Sphingomonas**	-.517*	.525*	.470*	-0,210	0,195	-0,026	-0,362	.513*	-.775**	.478*	0,418	1,000					
**Aspergillus**	0,414	-0,428	-0,455	0,064	-0,123	-0,341	0,098	-0,344	0,257	-.470*	0,011	-0,218	1,000				
**Exophiala**	-0,154	0,156	0,108	.599**	-.521*	0,171	-0,135	-0,106	-0,154	0,228	0,342	0,255	-0,160	1,000			
**Fusarium**	-0,040	0,057	0,038	0,026	0,003	-0,388	0,154	-0,187	0,236	0,049	-0,028	-0,356	0,416	-0,282	1,000		
**Penicillium**	-0,108	0,096	0,152	-0,362	0,317	0,110	-0,164	0,439	-0,412	0,065	0,098	.546*	-0,098	-0,030	-0,441	1,000	
**Rhodotorula**	-0,077	0,059	0,057	-.812**	.792**	-0,231	-0,439	.639**	-0,373	0,172	-0,131	0,430	-0,220	-0,342	-0,255	.501*	1,000

****. Correlation is significant at the 0.01 level (2-tailed).**
***. Correlation is significant at the 0.05 level (2-tailed).**
