# Supplementary material for: Influence of phosphate dosing on biofilms development on lead in chlorinated drinking water bioreactors
Source: NPJ Biofilms Microbiomes. 2020 Oct 23;6:43. doi: 10.1038/s41522-020-00152-w (PMC7585443; doi:10.1038/s41522-020-00152-w)
Supplement: Supplementary file 2 — Reporting Summary [file 41522_2020_152_MOESM2_ESM.pdf]

nature research

Corresponding author(s): Dr. Isabel Douterelo
NPJBIOFILMS-00742R

Last updated by author(s): Jul 18, 2020

Reporting Summary

Nature Research wishes to improve the reproducibility of the work that we publish. This form provides structure for consistency and transparency in reporting. For further information on Nature Research policies, see our Editorial Policies and the Editorial Policy Checklist.

Statistics

For all statistical analyses, confirm that the following items are present in the figure legend, table legend, main text, or Methods section.

n/a	Confirmed	
☐	☒	The exact sample size (*n*) for each experimental group/condition, given as a discrete number and unit of measurement
☐	☒	A statement on whether measurements were taken from distinct samples or whether the same sample was measured repeatedly
☐	☒	The statistical test(s) used AND whether they are one- or two-sided<br>*Only common tests should be described solely by name; describe more complex techniques in the Methods section.*
☐	☒	A description of all covariates tested
☒	☐	A description of any assumptions or corrections, such as tests of normality and adjustment for multiple comparisons
☐	☒	A full description of the statistical parameters including central tendency (e.g. means) or other basic estimates (e.g. regression coefficient) AND variation (e.g. standard deviation) or associated estimates of uncertainty (e.g. confidence intervals)
☐	☒	For null hypothesis testing, the test statistic (e.g. *F*, *t*, *r*) with confidence intervals, effect sizes, degrees of freedom and *P* value noted<br>*Give P values as exact values whenever suitable.*
☒	☐	For Bayesian analysis, information on the choice of priors and Markov chain Monte Carlo settings
☒	☐	For hierarchical and complex designs, identification of the appropriate level for tests and full reporting of outcomes
☒	☐	Estimates of effect sizes (e.g. Cohen's *d*, Pearson's *r*), indicating how they were calculated

Our web collection on statistics for biologists contains articles on many of the points above.

Software and code

Policy information about availability of computer code

Data collection: no software was used

Data analysis:
-MR DNA Molecular research analysis tool for sequencing analysis (http://www.mrdnalab.com/mrdnafreesoftware/)
-Venn diagrams were calculated using the web tool provided by the Bioinformatics & Evolutionary Genomics group at the University of Gent (http://bioinformatics.psb.ugent.be/webtools/Venn/).
-Alpha-diversity metrics were calculated with the software PAST version 4.0 [Hammer, Ø., Harper, D. A. T. & Ryan, P. D. PAST-palaeontological statistics software pakage for education and data analysis. Palaeontol. Electron. 4, 1–9 (2001)].
-Non-metric multi-dimensional scaling diagrams and Analysis of similarity statistics were done using Primer-E v7 (PRIMER-E, Plymouth, UK) [Clarke, K. & Warwick, R. Primer-6 computer program. (2005)].
-Non-parametric Mann-Whitney U test and Spearman's rank non-parametric correlations were done using SPSS Statistics 26 (IBM, USA).

For manuscripts utilizing custom algorithms or software that are central to the research but not yet described in published literature, software must be made available to editors and reviewers. We strongly encourage code deposition in a community repository (e.g. GitHub). See the Nature Research guidelines for submitting code & software for further information.

Data

Policy information about availability of data

All manuscripts must include a data availability statement. This statement should provide the following information, where applicable:
- Accession codes, unique identifiers, or web links for publicly available datasets
- A list of figures that have associated raw data
- A description of any restrictions on data availability

All data supporting the findings of this study are available from the corresponding author upon request.

Field-specific reporting

Please select the one below that is the best fit for your research. If you are not sure, read the appropriate sections before making your selection.

☐ Life sciences ☐ Behavioural & social sciences ☒ Ecological, evolutionary & environmental sciences

For a reference copy of the document with all sections, see nature.com/documents/nr-reporting-summary-flat.pdf

Ecological, evolutionary & environmental sciences study design

All studies must disclose on these points even when the disclosure is negative.

Study description

This research aims to study the effect of different doses of phosphate (below 1 mg/L, 1 mg/L and 2 mg/L) on biofilms grown in reactors with lead and PVC supports, and fed with drinking water.

Research sample

The samples consisted in biofilms grown for 28 day in lead coupons (1.27 cm of diameter) and black PVC flexible tubing (inner diameter: 9.75mm, length: 75cm). Also water samples were collected following the instructions of ALS Environmental (Coatbridge, UK)

Sampling strategy

All samples were taken in triplicate. Due to the amount of biofilm grown, more than three replicates would supposed less DNA to analysed and with low quality. for water samples, due to the volume of the tanks, more replicates would implied a drastic reduction of the water level, and biofilms would dryed. Three replicates is an standart procedure in science and the authors considered it adecuated.

Data collection

All data collection was done by Gonzalo Del Olmo, ALS Environmental (Coatbridge, UK) and MR DNA Molecular Research Laboratory (www.mrdnalab.com, Texas, USA).
-Physicochemistry analysis: During the 28 days of the experiment , several physicochemical factors were analysed daily for the water in the three DWBR as well as the local drinking water feeding them. Every analysis was performed in triplicate and the average of the replicates was calculated. Several parameter were measured daily in the laboratory; turbidity by means of a Palintest turbidity meter (Palintest PTH7091, UK), chlorine (free and total) with a chlorine meter (ChloroSense Palintest, UK), temperature and pH using a HannaH1991003 pH-meter (Hanna Instruments, Bedfordshire, UK) and orthophosphate concentration was measured as explained above with PhosVer® using a Jenway 7300 Visible spectrophotometer. In addition, triplicate bulk water samples were collected weekly, and analysed for total lead, total iron, orthophosphate, total phosphorus and total organic carbon by an accredited drinking water laboratory, ALS environmental (Coatbridge, UK).
-Biofilm sampling: After 28 days of biofilm development, lead coupons were collected from the bioreactor and placed in a sterile petri dish with 30 ml of Phosphate-buffered saline (PBS) (pH 7).Then coupons were brushed using toothbrushes to remove the biofilm attached to the coupon surface as described in Deines et al. (2010)87. The toothbrushes were previously sonicated in an Ultrasonic Water Bath (Model AL04-04-230, Advantage-Lab, Menen, Belgie) during 45 min in a solution of 2% of RBS and during 10 min in distilled water, and then, they were sterilized in an autoclave (Autoclave Prestige 2100 Classic 9 litres, Prestige Medical, Blackburn, UK) during 20 min at 121 °C. The biofilm solutions were filter through a sterile filter (0.22 µm MCE Membrane MF-Millipore, UK), and the filters were kept at -20 °C until DNA extraction was performed. In addition to the lead coupons, 3 pieces of PVC flexible tubing of 75 cm from each bioreactor were taken after 28 days to study biofilm growth on this material. A longitudinal cut along the tubes was performed with a sterile scalpel, and then, each half tube was brushed internally using sterile toothbrushes and 30 mL of PBS (pH 7). The PBS solutions with the biofilms were filter through a sterile filter (0.22 µm MCE Membrane MF-Millipore, UK), and filters kept at -20 °C until DNA extraction. After DNA extraction, samples were sequences by MR DNA Molecular Research Laboratory and analysed acording it own pipeline.

Timing and spatial scale

The experiment started the 30th of October (2019) and ended the 27th of November (2019). Water parameters analysed in the lab were taken daily, while parameters analysed by ALS were taken every 7 days. Biofilms samples were taken at the end of the experiment only (28 days)

Data exclusions

No data were excluded

Reproducibility

All samples were taken in triplicate as it was mentioned before

Randomization

This is not relevant for this study. This research did not work with groups of individuals.

Blinding

This is not relevant for this study. As mentioned before, this research did not work with groups of individuals.

Did the study involve field work? ☐ Yes ☒ No

Reporting for specific materials, systems and methods

We require information from authors about some types of materials, experimental systems and methods used in many studies. Here, indicate whether each material, system or method listed is relevant to your study. If you are not sure if a list item applies to your research, read the appropriate section before selecting a response.

Materials & experimental systems

n/a	Involved in the study
☒	☐ Antibodies
☒	☐ Eukaryotic cell lines
☒	☐ Palaeontology and archaeology
☒	☐ Animals and other organisms
☒	☐ Human research participants
☒	☐ Clinical data
☒	☐ Dual use research of concern

Methods

n/a	Involved in the study
☒	☐ ChIP-seq
☒	☐ Flow cytometry
☒	☐ MRI-based neuroimaging
